# Supplementary material for: Hopping and Flipping of RNA Polymerase on DNA during Recycling for Reinitiation after Intrinsic Termination in Bacterial Transcription
Source: Int J Mol Sci. 2021 Feb 27;22(5):2398. doi: 10.3390/ijms22052398 (PMC7957599; doi:10.3390/ijms22052398)
Supplement: Supplementary file 1 [file ijms-22-02398-s001.pdf]

Hopping and flipping of RNA polymerase on DNA during recycling for reinitiation after intrinsic termination in bacterial transcription

Wooyoung Kang[1], Seungha Hwang[2], Jin Young Kang[2], Changwon Kang[3]* and Sungchul Hohng[1]*

[1] Department of Physics and Astronomy, and Institute of Applied Physics, Seoul National University, Seoul 08826, Republic of Korea; crn1860@gmail.com (W.K.)

[2] Department of Chemistry, Korea Advanced Institute of Science and Technology, Daejeon 34141, Republic of Korea; tmdgk.hwang@kaist.ac.kr (S.H.); jykang59@kaist.ac.kr (J.Y.K.)

[3] Department of Biological Sciences, Korea Advanced Institute of Science and Technology, Daejeon 34141, Republic of Korea.

***** Correspondence: ckang@kaist.ac.kr (C.K.); shohng@snu.ac.kr (S.H.)

Supplementary Figures

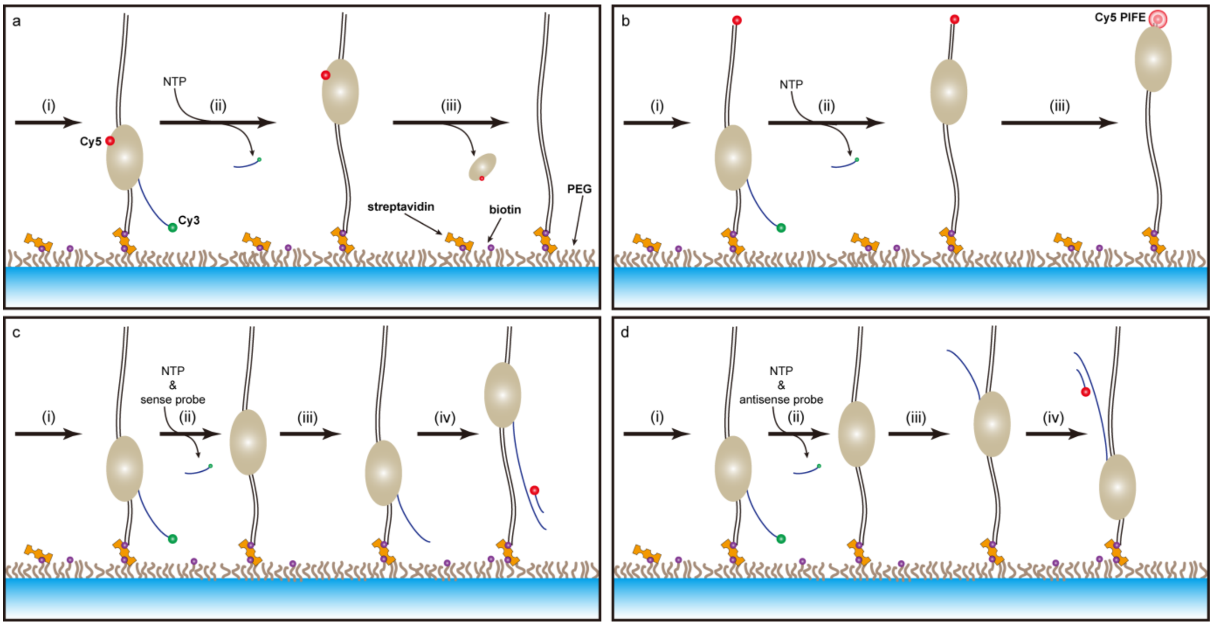

Figure S1. Experimental schemes. (a) Measurements of RNAP retention time in Figure 1. Early transcription complexes with Cy3-RNA and Cy5-RNAP are stalled and immobilized on slides by biotin-streptavidin conjugation before extensively washed (step i). When elongation is resumed by NTP addition, sometime after Cy3-RNA is released at termination (step ii), recycling Cy5-RNAP dissociates from DNA (step iii). (b) Measurements of 1D diffusion coefficient of recycling RNAP in Figure 2. The scheme is the same as (a), except that recycling RNAP reaches the downstream end of DNA and causes Cy5 PIFE (step iii). (c) Sense reinitiation experiments in Figure 3. The scheme is the same as (a), except that recycling RNAP reinitiates sense transcription (step iii) and that the Cy5-labeled sense probe binds sense transcripts (step iv). (d) Antisense reinitiation experiments. The scheme is the same as as (a), except that the Cy5-labeled antisense probe binds antisense transcripts (steps iii and iv).

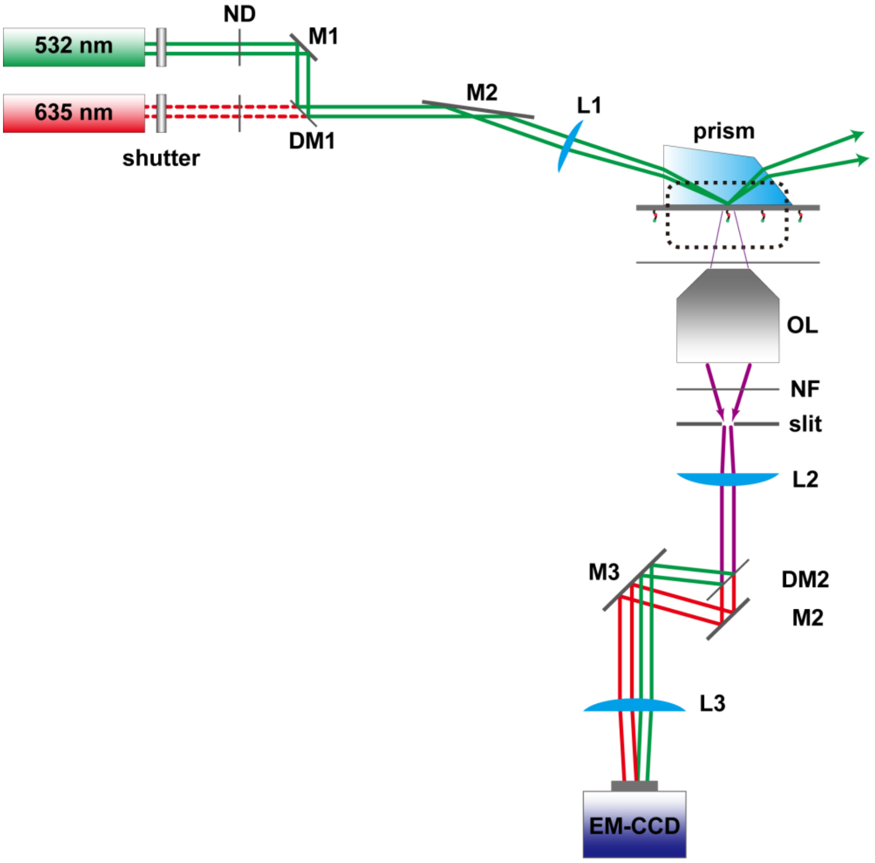

Figure S2. A schematic diagram of the total-internal-reflection fluorescence microscope. The abbreviations used are L for lens, M for mirror, DM for dichroic mirror, OL for objective lens, and NF for notch filter.

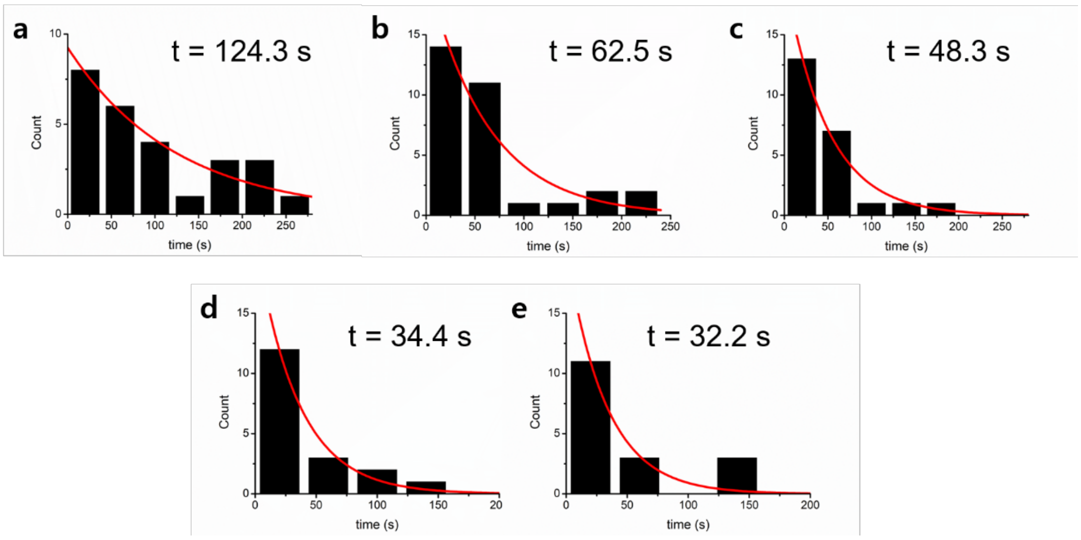

Figure S3. Retention time of recycling RNAP at varying salt concentrations. We measured the time difference $t_{retention}$ between the Cy3 signal vanishing and Cy5 signal vanishing at NaCl concentrations varying from 20 mM (n = 26) (a), 50 mM (n = 31) (b), 100 mM (n = 26) (c), 150 mM (n = 18) (d) to 200 mM (n = 17) (e). To obtain the retention times, the distributions were fitted to a single-exponential decay function.

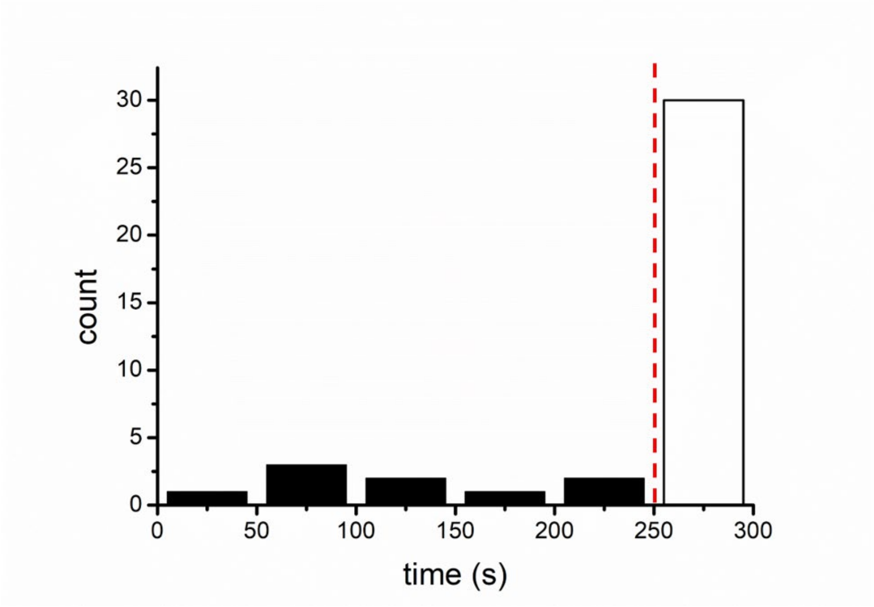

Figure S4. Distribution of Cy5 photobleaching time. We performed the same experiments as the RNAP retention experiments except that NTPs were not added. From 39 molecules observed, 70% of molecules (white bar) survived for 250 s after starting the experiments. By assuming that photobleaching is a single-step process whose survival probability is expressed as $e^{-t/\tau}$, the photobleaching time is estimated as 750 (= -250/ln 0.7) s.

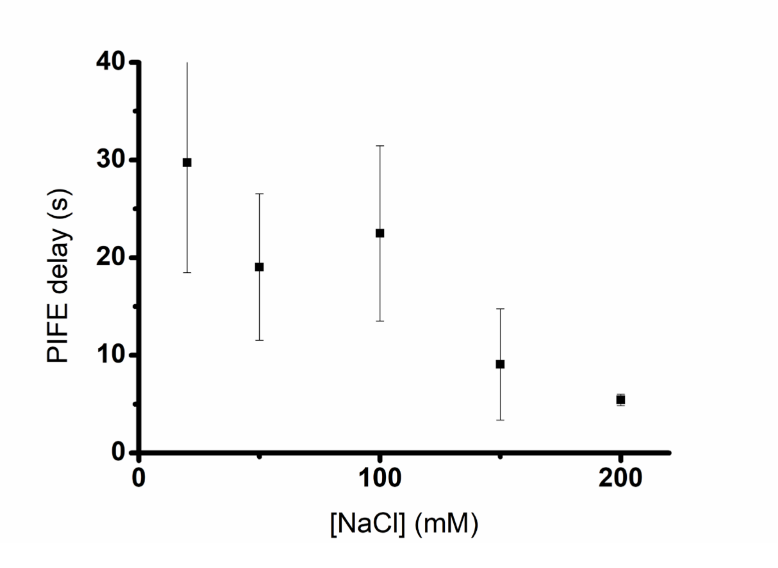

Figure S5. PIFE delay plotted against NaCl concentration for DNA template L+212. PIFE delay tends to decrease with rising NaCl concentrations from 20 to 200 mM, suggesting faster diffusion at higher salt concentrations.

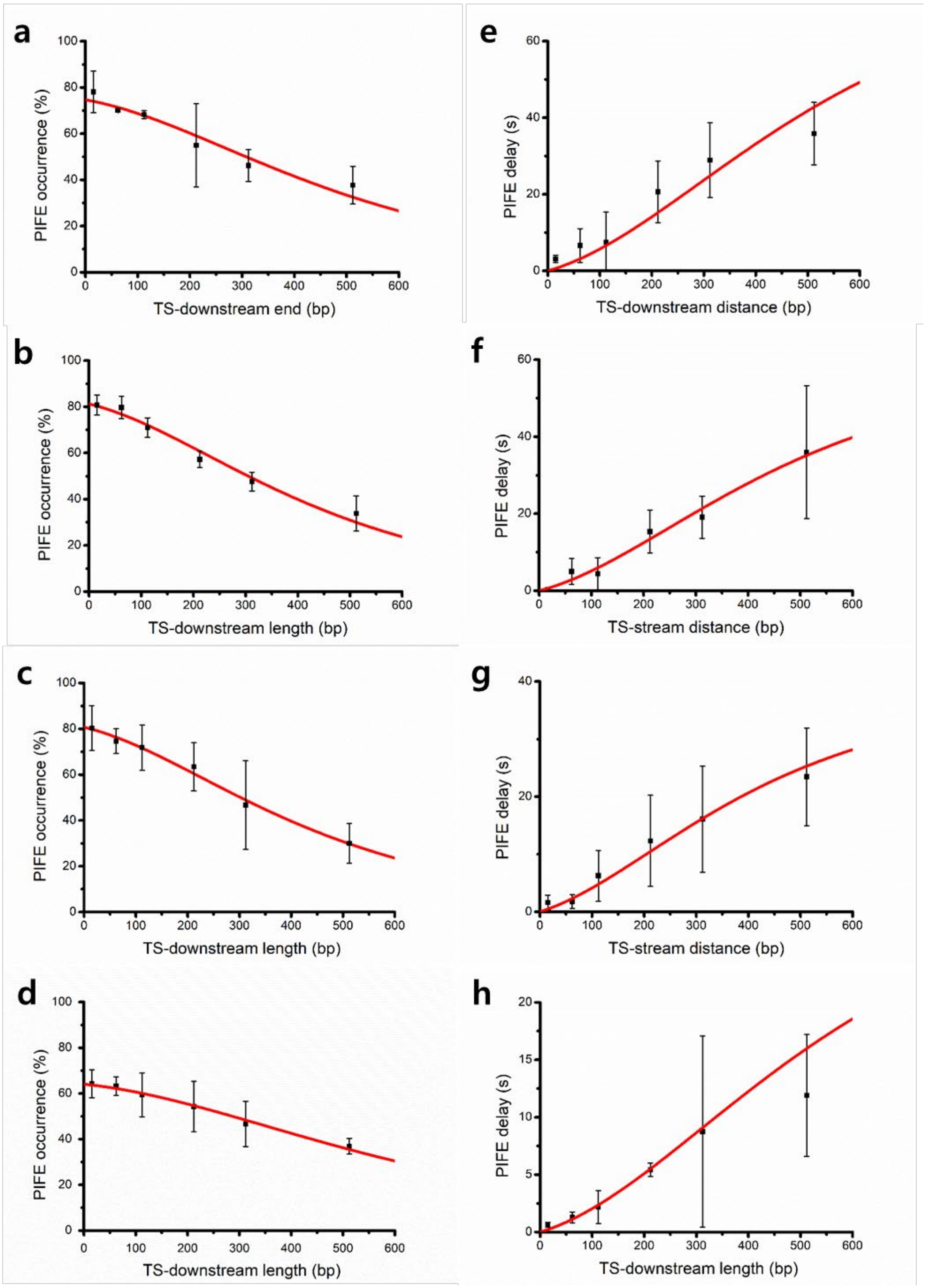

Figure S6. PIFE occurrence and delay at varying NaCl concentration. (a-d) PIFE occurrences are plotted as a function of TS-downstream length for the data from the experiments with 20 mM (a), 50 mM (b), 100 mM (c) and 200 mM (d) NaCl. (e-h) PIFE delays are plotted as a function of TS-downstream length for the data from the experiments with 20 mM (d), 50 mM (e), 100 mM (f) and 200 mM (h) NaCl. All data are fitted to a 1D diffusion model described in Materials and Methods (red line). Additionally, both PIFE occurrence and delay data at 150 mM NaCl are shown in Figure 2e,f.

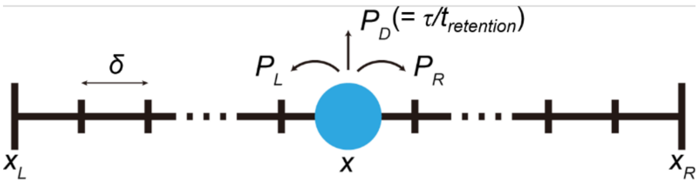

Figure S7. A model for 1D diffusion of recycling RNAP on DNA. Linear duplex DNA is depicted as a straight line with a finite length. The left-side end of DNA is an immobilized reflecting end on surface side, and the right-side end is an absorbing end on buffer side. RNAP is assumed to make 1D random walk motions each with a step size δ for a time interval τ. For every step of a random walk, RNAP can dissociate with a probability of $P_D = \tau/t_{retention}$, where $t_{retention}$ is the measured lifetime of RNAP bound on DNA (Figure 1). Therefore, the probability of movement to the left (P_L) or right (P_R) can be expressed as $1/2(1 - \tau/t_{retention})$.

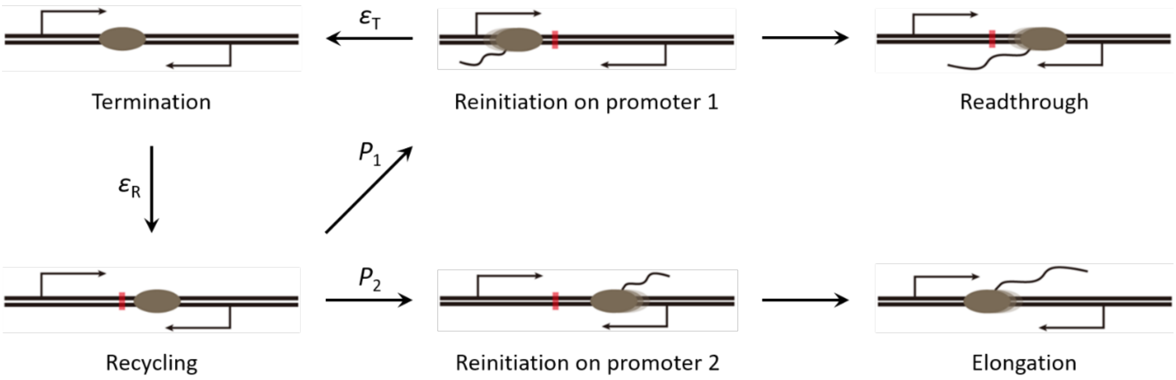

Figure S8. A cycle of termination, recycling, and reinitiation. On DNA template L+2P, downward (rightward) transcription from promoter 1, but not upward (leftward) transcription from promoter 2, terminates at TS (red vertical line) with termination efficiency, ε_T. After transcript is released at TS, RNAP mostly (recycling efficiency, ε_R) remains on DNA entering the recycling stage, where RNAP one-dimensionally diffuses in downward and upward directions with occasional flipping. Although RNA-free RNAP can exit the recycling stage by falling off DNA or get inactivated on DNA at any time (not shown), recycling RNAP can render reinitiation on promoter 1 or 2 with probability P_1 or P_2, respectively. After reinitiation on promoter 1, RNAP can repeat the cycle of termination at TS, recycling on DNA, and another reinitiation on promoter 1, until a readthrough at TS, a reinitiation on promoter 2, or an exit from recycling breaks the cycle.

DNA sequences

Supplementary Table S1. Template DNA sequences

Oligonucleotide name	Sequence (5' to 3')
UP8_template	TATCA AAAAG AGTAT TGACT TAAAG TCTAA CCTAT AGGAT ACTTA CAGCC ATCGA ACAGG CCTGC TGGTA ATCGC AGGCC TTTTT ATTTG GGGGA GAGGG AAGTC ATGAA AAAAC TAACC TTTGA AATTC GATCT CCAGG ATCCA CCACC
UP8_template_2	GCGAG ATTAC CATTA AGTGA ATTCG AAAAA AGCAC GCTAC CGCCC CAGGC GGTGG TGGAT CCTGG AGATC GAATT TCAAA GGTTA GTTTT TTCAT GACTT CCCTC TCCCC CAAAT AAAAA GGCCT GCGAT TACCA GCAGG CCTGT TCGAT GGCTG TAAGT ATCCT ATAGG TTAGA CTTTA AGTCA ATACT CTTTT TGATA
additional_part	pAATTC TTACA ATTTA GACCC TAATA TCACA TCAGA CACTA ATTGC CTCTG CCAAA ATTCT GTCCA CAAGC GTTTT AGTTC GCCCC AGTAA AGTTG TCAAT AACGA CCACC AAATC CGCAT GTTAC GGGAC TTCTT ATTAA TTCTT TTTTC GTGGG GAGCA GCGGA TCTTA ATGGA TGGCG CCAGG TGGTA TGGAA GC
additional_part_2	pGGGCT GAAAG TAGCG CCGGG TAAGG TACGC GCCTG GTATG GCAGG ACTAT GAAGC CAATA CAAAG GCTAC ATCCT CACTC GGGTG GACGG AAACG CAGAA TTATG GTTAC TTTTT GGATA CGTGA AACAT GTCCC ATGGT AGCCC AAAGA CTTGG GAGTC TATCA CCCCT AGGAC ACACA AGACA CCACA AGCTT AGACC
DNA_splint	TGTGA TATTA GGGTC TAAAT TGTAA GAATT GCGAG ATTAC CATTA AGTGA ATTCG AAAAA
DNA_splint_2	GCGTA CCTTA CCCGG CGCTA CTTTC AGCCC GCTTC CATAC CACCT GGCGC CATCC ATTAA
L+2P_part1	ACTAT CTATT CTCCC ATCTA TCAAA AAGAG TATTG ACTTA AAGTC TAACC TATAG GATAC TTACA GCCAT CGAAC AGGCC TGCTG
L+2P_part2	GTGGA ATTCA CTTAA TGTGT GTGGT CTGTG GTGTC TTGTG TGTGG TCTGT GGTGT CTTGT GTGTG GTCTG TGGTG TCTTG TGTGT GGTCT GTGGT GTCTT GTGTG TGGTC TGTGG TGTCT GCAGG CTGTA AGTAT CCTAT AGGTT AGACT TTAAG TCAAT ACTCT TTTTG ATACA CTGCG CGATA CATAA GCTTC GACGT
L+2P_splint	ACACA TTAAG TGAAT TCCAC GCGAG ATTAC CATTA AGTGA A

Supplementary Table S2. Primer sequences

Oligonucleotide name	Sequence (5' to 3')
forward_primer_biotin	Biotin-TATCA AAAAG AGTAT TGACT TAAAG TC
reverse_primer_L+15	Cy5-CTTCC CTCTC CCCCA AATAA AAAG
reverse_primer_L+62	Cy5-GGTGG TGGAT CCTGG AGATC G
reverse_primer_L+112	Cy5-GCGAG ATTAC CATTA AGTGA A
reverse_primer_L+212	Cy5-GACAA CTTTA CTGGG GCGAA CTAAA AC
reverse_primer_L+312	Cy5-GCTTC CATAC CACCT GGCGC CATCC AT
reverse_primer_L+512	Cy5-GGTCT AAGCT TGTGG TGTCT TGTGT GT
lambda_forward_primer	GTTTT CTGGG TTGGT
lambda_reverse_primer	GGCGG GTTTT GTTTT
forward_primer_biotin_α	Biotin-ACTAT CTATT CTCCC ATC
Reverse_primer_L+2P	ACGTC GAAGC TTATG TATCG CGCAG TG
